# Beat-to-Beat QT Variability: A Population Study of the QT Variability Index Composition

**DOI:** 10.3390/diagnostics16030502

**Published:** 2026-02-06

**Authors:** Jan Řehoř, Kateřina Helánová, Martina Šišáková, Tomáš Novotný, Irena Andršová, Marek Malik

**Affiliations:** 1 Department of Internal Medicine and Cardiology, University Hospital Brno, Jihlavská 20, 625 00 Brno, Czech Republic; rehor.jan@fnbrno.cz (J.Ř.); sisakova.martina@fnbrno.cz (M.Š.); novotny.tomas3@fnbrno.cz (T.N.); andrsova.irena@fnbrno.cz (I.A.); 2 Department of Internal Medicine and Cardiology, Faculty of Medicine, Masaryk University, Jihlavská 20, 625 00 Brno, Czech Republic; 3 National Heart and Lung Institute, Imperial College, 72 Du Cane Rd, Shepherd’s Bush, London W12 0NN, UK; marek.malik@imperial.ac.uk

**Keywords:** QT variability index, healthy subjects, postural testing, heart rate variability, multivariable regression

## Abstract

**Background/Objectives**: One of the topics of electrocardiographic risk factor studies is investigations of beat-to-beat QT interval variability. The seminal study that reported QT variability as a prognostic risk factor introduced the so-called QT variability index (QTVi). QTVi quantification relies not only on the variance of QT intervals but also on correction factors, including RR interval variance, heart rate, and overall QT interval duration. This study investigated the influence of all the measured factors on QTVi values. **Methods**: Long-term electrocardiograms (ECGs) were obtained from 251 healthy subjects (mean age 33.6 ± 9.1 years, 108 females) during repeated postural tests that involved supine, sitting, and standing positions maintained for 10 or 15 min. During each position, a 5-min ECG segment with a stable heart rate and without any ectopic disturbances was found. In these segments, standard deviations of normal-to-normal RR (NN) interval durations (SDNN) and of beat-to-beat QT interval durations (SDQT) were measured together with the means of NN and QT intervals. QTVi was subsequently calculated. For each subject, results obtained during each postural position were averaged. **Results**: In multivariable regression models, evaluated separately in female and male sex-subgroups of the population, QTVi values were significantly dependent on SDQT, SDNN, and mean NN intervals (all *p* < 0.001) but practically independent of mean QT interval durations. **Conclusions**: QTVi is significantly influenced by factors that are unrelated to the beat-to-beat changes in QT interval durations. This needs to be considered when interpreting QTVi values. In future studies, multivariable statistical models are needed to ensure that QTVi findings are independent of associated heart rate variability indices.

## 1. Introduction

Electrocardiography is one of the simplest medical investigations providing objective data. Indeed, the standard clinical electrocardiogram (ECG) is regularly collected in the majority of all patients regardless of whether they do or do not suffer from cardiovascular abnormalities or diseases and regardless of whether they are seen by general practitioners, investigated in specialised ambulatory clinics, or hospitalised.

In addition to the well-known diagnostic use of clinical ECG recordings, different possibilities of prognostic estimates have also been repeatedly proposed, utilising characteristics that are beyond visual ECG diagnostics. Rather recently, prognostic methods have been based on artificial intelligence models [1,2,3], but more experience exists with prognoses based on ECG morphology [4,5] and beat-to-beat changes [6,7,8]. The category of risk prediction based on beat-to-beat changes is represented mainly by investigations of heart rate variability (HRV) that have been, over the last decades, performed in a very wide variety of clinical populations ranging from patients with ischaemic heart disease [9], hypertension [10,11], diabetes [12], psychiatric problems [13], and many other conditions ranging from critical medicine [14] and primary medical care [15] to studies in the general overall population [16,17]. In addition to HRV, beat-to-beat variations in other ECG measurements and indices have also been studied. Because of the known relationship between repolarization abnormalities and arrhythmogenesis, a number of studies have concentrated on the variability and abnormality of T wave morphologies and of the QT interval durations. One of the initial attempts to quantify T wave abnormality was the concept of QT dispersion [18] that was subsequently recognised as ill-founded [19,20]. In terms of beat-to-beat repolarisation variability, T wave alternans methods concentrate on one-to-one periodic changes in the T wave vector orientation and amplitude [21]. Many other investigations of QT interval variability (QTV) researched both periodic and aperiodic variations in the interval durations. Similarly to HRV-based risk prediction, studies of QTV-based risk stratification have been reported in a broad variety of clinical populations [22,23,24].

As previously reviewed [25], QTV studies use a variety of indices expressing the variability of the QT interval durations numerically. The majority of these studies have followed the suggestion in the seminal publication and used the so-called QTV index [26]. As described in more detail in the Methods section, this index is a composite measure that incorporates not only the variance of QT interval duration but also the variance of underlying heart rate, mean QT interval duration, and mean heart rate of the analysed ECG recording. The seminal article that introduced the QTV index suggested that the index normalises the absolute QTV by the degree of heart rate variability observed in the analysed ECG. This construct thus suggests that QTV is influenced by HRV in a way conceptually similar to the dependency of QT interval duration on the underlying heart rate, i.e., that simple direct expressions of QTV (such as QT interval variance) require correction for underlying HRV to make them independent of HRV in a similar way as the QTc intervals are, ideally, independent of heart rate. The practical as well as physiological validity of the QTV index (QTVi) thus depends on the relationship between the index and the factors for which the index is normalised.

Having this in mind, we have conducted a physiological study in a population of healthy subjects to investigate not only the relationship between the QTVi and its correction factors but also between the individual constituents of QTVi.

## 2. Materials and Methods

### 2.1. Definition of QT Variability Index

As initially proposed [26], the QTV index (QTVi) is defined as

$$QTVi=\log\left(\frac{\;\;\;\;\;\frac{{QT}_{var}}{{\left({QT}_{mean}\right)}^{2}}\;\;\;\;\;}{\;\;\;\;\;\frac{{HR}_{var}}{{\left({HR}_{mean}\right)}^{2}}\;\;\;\;\;}\right)$$

where ${QT}_{var}$ and ${HR}_{var}$ mean variances of QT interval duration and of heart rate, respectively, ${QT}_{mean}$ and ${HR}_{mean}$ are the mean values of QT interval duration and of heart rate, and log is a decadic logarithm. Since the concept of beat-to-beat heart rate variance is somewhat unusual, many studies have replaced it with RR interval variance and used the formula

$$QTVi=\log\left(\frac{\;\;\;\;\;\frac{{QT}_{var}}{{\left({QT}_{mean}\right)}^{2}}\;\;\;\;\;}{\;\;\;\;\;\frac{{RR}_{var}}{{\left({RR}_{mean}\right)}^{2}}\;\;\;\;\;}\right)$$

where ${RR}_{var}$ and ${RR}_{mean}$ mean the variance and mean of RR interval durations. (We shall subsequently show that in our data, this formula change led to only minimal numerical differences.) The variance is the square of the standard deviation; thus, using elementary simplifications of logarithmic expressions:

$$QTVi=\;\log\left(\frac{\;\;\;\frac{{SDQT}^{2}}{{\left({QT}_{mean}\right)}^{2}}\;\;\;}{\;\;\;\frac{{SDRR}^{2}}{{\left({RR}_{mean}\right)}^{2}}\;\;\;}\right)\;=\log\left(\frac{\;\;{\left(\;\frac{SDQT}{{QT}_{mean}}\right)}^{2}\;\;\;}{\;\;\;{\left(\frac{SDRR}{{RR}_{mean}}\right)}^{2}\;\;\;}\right)=2\;\log\left(\frac{\;\;\;\;\;\frac{SDQT}{{QT}_{mean}}\;\;\;\;\;}{\;\;\;\;\;\frac{SDRR}{{RR}_{mean}}\;\;\;\;\;}\right)\;=2\;\log\left(\frac{SDQT}{SDRR}\times \frac{{RR}_{mean}}{{QT}_{mean}}\right)\;=2\;\left(\log\left(SDQT\right)+\log\left({RR}_{mean}\right)-\;\log\left(SDRR\right)-\log\left({QT}_{mean}\right)\right)$$

where *SDRR* and *SDQT* are standard deviations of the RR and QT interval durations, respectively. Exactly the same manipulations are applicable to the formula in which QTVi is corrected for the proportion between the heart rate variance and the mean heart rate.

Based on these properties of the definition of the QTV index, we have investigated the relations between the elements constituting the index. Intentionally, we used a dataset that included different autonomically active stages, i.e., stages with known differences between HRV and heart rate.

### 2.2. Study Population

The data analysed in this investigation were obtained during a previously described large clinical pharmacology study conducted in healthy subjects [27,28]. Repeated 12-lead daytime Holter recordings were made in all study subjects while they were on no treatment. During the complete clinical pharmacology study, the subjects were free of alcohol and/or caffeinated drink ingestion. All subjects had a normal clinical ECG and normal clinical investigations during the screening phase, as usual in clinical pharmacology studies [29]. Female subjects of the study had a negative pregnancy test and, for the duration of the clinical pharmacology study, were not on hormonal contraceptives. Age and other demographic descriptions were recorded at study enrolment.

The original study was conducted at two different centres and was approved by the relevant ethics boards (Parexel, Baltimore, MD, USA; and California Clinical Trials, Glendale, CA, USA). All participants provided written informed consent. The consent included the possibility of additional future investigations of the research data. Both the formulation of the informed consent and all characteristics of study conduct fully satisfied the requirements of the Helsinki declaration. Since only anonymised off-treatment data are presented here, further details of the source study are of no relevance.

### 2.3. Baseline Investigations

Drug-free (that is, both active-compound-free and placebo-free) long-term ECG recordings were obtained at 4 different study days within a 4-week period [27]. On each of these days, 2 separate sequences of postural provocations were incorporated into the study protocol. During the first sequence, a 10-min strict supine position was followed by a 10-min unsupported sitting position, followed by a 15-min unsupported standing position, followed by a 10-min strict supine position. During the second sequence, the order of the standing and sitting positions was inverted; that is, a 10-min strict supine position was followed by a 15-min standing position, followed by a 10-min sitting position, followed by a 10-min strict supine position. Study subjects performed these postural position changes themselves without any external support apart from verbal instructions. During the provocative tests, the study subjects were not allowed to speak and made no bodily movements apart from shallow breathing. No external disturbances were allowed during these provocative position tests.

### 2.4. ECG Recordings and Measurements

Continuous 12-lead Holter recordings were obtained during the study days and covered the postural provocative tests. Using previously described measurement procedures [30,31] individual QRS complexes were identified and classified as belonging to sinus rhythm or corresponding to supraventricular or ventricular ectopic beats. A series of RR intervals measured at a 1 kHz resolution was obtained with systematic timing of QRS complexes achieved by calculating their cross-correlation maxima over individual leads.

The recordings were divided into 10 s segments with 5 s overlap between neighbouring segments. In each of these, a representative beat form was constructed for each ECG lead using median values around individual QRS complexes synchronised by their timing within the 10 s segment. The beat forms of all ECG leads were superimposed on the same isoelectric axis. In each ECG recording of a postural provocative test, at least 5 non-overlapping and non-adjacent ECG segments were selected within the period of each postural position. Using previously described measurement and quality control methods [30,31], QRS onset and T wave offset were identified and validated in these selected representative beat forms, including the assurance of measurement systematicity, i.e., assurance that similar ECG patterns were interpreted and measured in the same way [32]. Subsequently, using a cross-correlation method for pattern identification and replicating the methodology previously proposed [26], these measurements were projected to representative beat forms of all ECG segments recorded during the postural provocative test. The same cross-correlation technique [26] was subsequently employed to obtain the QRS onset and T wave offset in all cardiac cycles. The overlap between the 10 s segments allowed for ensuring that no abrupt measurement shifts existed between individual segments. Repeated visual control of independently selected segments was subsequently used to verify the validity of the ECG measurements at the level of individual beats. In this way, sequences of QT interval durations were obtained and associated with the measured RR intervals. The measurements were performed with 1 millisecond (ms) precision.

### 2.5. Electrocardiographic Data Evaluation

Data from all postural provocative tests were excluded from the analysis if the subject was unable to complete any of the tests because of pre-syncopal symptoms, substantial discomfort, or other reasons that prevented the test completion. All data of a subject were also excluded from the analysis if the subject dropped out of the study before or during the fourth baseline day. Data were further excluded if the cross-correlation measurement of the QT interval was compromised because of excessive noise (e.g., due to disconnected leads). In each subject and in each postural position, a 5 min sub-interval was selected with minimal heart rate spread (i.e., minimal difference between heart rates measured in 30-second intervals). If it were impossible to find such sub-intervals without any ventricular or supraventricular ectopic beats for all postural positions in a subject, the data of the subject were excluded.

Thus, in each subject eligible for this analysis, we identified ectopic-free 5-min sub-intervals in all 8 postural episodes of each of the 4 drug-free baseline days. In every such sub-interval, we calculated the mean normal-to-normal RR interval in ms, the mean QT interval duration in ms, the standard deviation of all normal-to-normal (NN) RR intervals (SDNN), and the standard deviation of all QT intervals (SDQT). To satisfy the original definition of QTVi, we also assigned a corresponding (i.e., single beat) heart rate value (in bpm) to each NN interval (based on the calculation Heart-rate = 60,000/NN). This allowed us to calculate the mean heart rate and the standard deviation of individual heart rate values (SDHR). Using these elementary measurements, we calculated their proportions, including the heart-rate-based and RR-interval-based expressions of the QTV index. We shall denote these indices QTVi_HR_ and QTVi_RR_ for the QTV index based on correction for SDHR and mean heart rate and based on correction for SDNN and mean RR interval, respectively.

### 2.6. Data Presentation and Statistics

In each study subject, ECG data obtained in each of the 8 different positions per day were firstly averaged over all 4 drug-free days and subsequently averaged over all supine, sitting, and standing positions. This provided, for each subject and for each measured index, 8 sequential values corresponding to the conducted tests of the postural changes, and further 3 overall averaged values corresponding to supine, sitting, and standing positions. The context of the data presentation shows clearly which of the averages are being analysed. Continuous data are presented as mean ± standard deviation (SD). The measured and derived ECG data are presented in an overall table and, where appropriate, repeated in the text. In the overall tabulated data, the QT interval durations were corrected for the underlying heart rate using the Fridericia formula [33,34].

Group comparisons (e.g., comparisons between females and males) were based on a two-sample two-tailed *t*-test assuming different variances between compared groups and accompanied by estimates of strictly standardised mean differences [35]. Intra-subject comparisons were based on a paired two-tailed *t*-test. Dependencies between different ECG indices were based on linear regressions. These are further displayed graphically with a distinction between female and male sub-populations and showing 95% percentiles of the regression slopes. Numerical values of regression slopes are provided, where appropriate, after normalisation for standard deviation (that is, the so-called β-coefficients are shown).

To evaluate the influence of elementary ECG measurements on the QTV index, multivariable linear regressions were used. That is, QTVi_RR_ was studied as a linear combination of SDQT, SDRR, mean QT intervals, and mean NN intervals. In the same way, QTVi_HR_ was studied as a linear combination of SDQT, SDHR, mean QT interval, and mean heart rate.

ECG processing and calculations of the ECG indices were made using internally validated packages developed in C++ (Microsoft Visual Studio 2022, 64-bit version 17.14.14) and Python (64-bit version 3.12.4), and statistical comparisons were made using the SPSS package (IBM, version 29.0.0.0). *p*-values below 0.05 were considered statistically significant. To compare the statistical significances, test values are presented where appropriate to avoid minuscule *p*-values that seem less seemly for conceptual interpretations.

Because of the interdependence between the analysed indices, no adjustment for multiplicity of statistical testing was employed. For instance, the evaluation of SDQT/SDNN, SDQT/(mean QT), SDNN/(mean NN), etc., relationships cannot be considered as testing independent datasets. Similar considerations apply to studies of QTVi dependency on different elementary measurements.

## 3. Results

### 3.1. Population and Electrocardiographic Data

The original clinical pharmacology study enrolled 176 females and 176 males. Of these, 33 subjects (9.4%) were prematurely discontinued. After applying the described exclusion criteria (see Section 2.4 and Section 2.5), the present study excluded a further 68 subjects (19.2%). These exclusions were due to ECG recording problems (missing ECG leads and/or excessive noise that did not allow valid projections of manually verified ECG measurements). The present study thus analysed ECG data of 251 subjects. Of these, 108 were females aged 34.2 ± 10.2 years, and 143 were males aged 33.2 ± 8.2 years (no significant difference between the ages of the sex groups).

The measured ECG data and their comparison between females and males are summarised in Table 1.

### 3.2. Comparison of QTV Index Versions

While the QTVi_RR_ values were significantly different between sexes in the sitting and standing positions, the sex difference was not significant in the supine position.

Supplementary Figure S1 shows comparisons between QTVi_RR_ and QTVi_HR_ in the three different positions. Although the differences between the index variants were small in single percentage figures of the index values, they were statistically significant, albeit inconsistently. In supine and sitting positions, the values of QTVi_RR_ were significantly higher than the QTVi_HR_ values, but in the standing position, the relationship reversed, and the QTVi_HR_ values were significantly higher than the QTVi_RR_ values. These observations applied to both sexes. Note also the regression slopes that show that the differences between the two QTVi variants were slightly biased, particularly in the supine and sitting positions. These results are possibly counterintuitive because of the mathematically strict relationship between NN interval durations and corresponding single-beat heart rate values. Note that this strict mathematical relation does not apply to the mean and variance values (the average and the variance of reciprocal values are not strictly equal to the reciprocal values of the average and of the variance).

### 3.3. Relationship Between Standard Deviations and Mean Values

**Figure 1:**
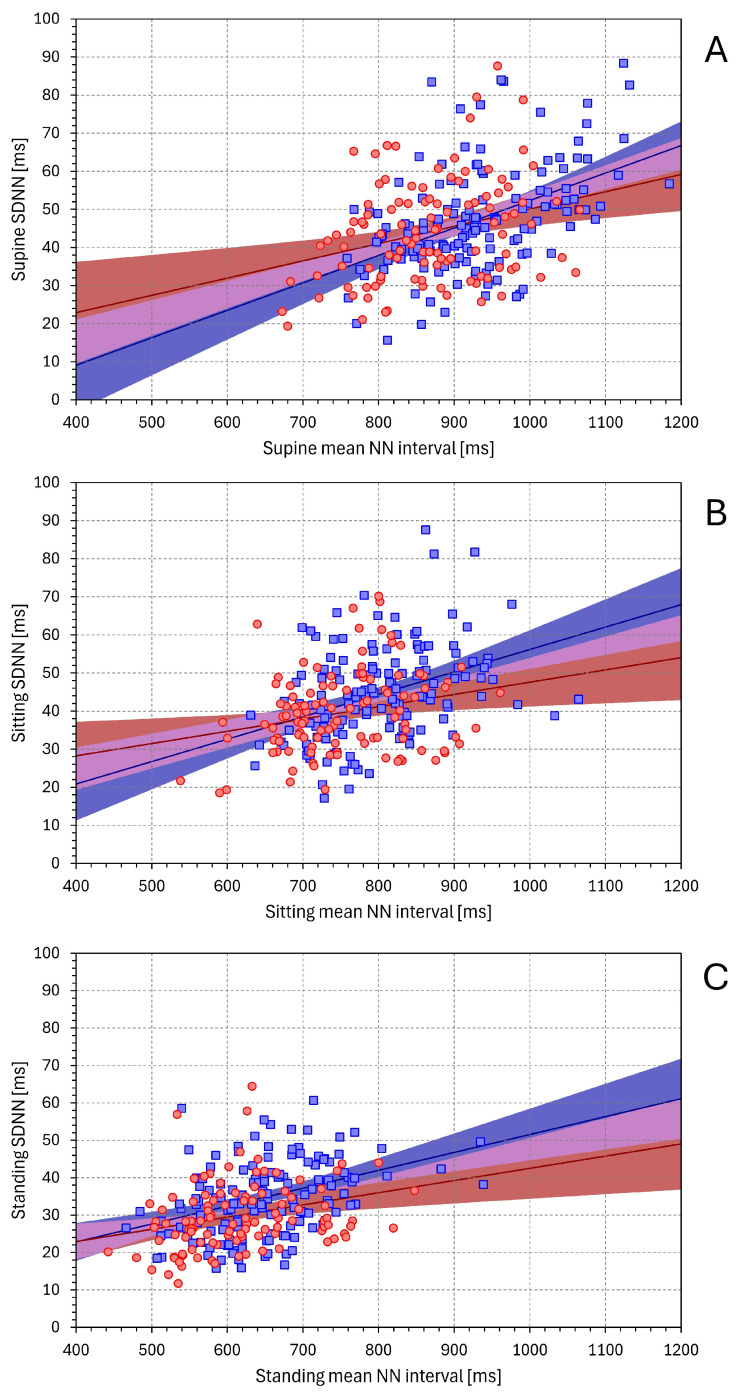
The relationship between mean NN interval duration and SDNN values in supine, sitting, and standing positions is shown in panels (**A**), (**B**), and (**C**), respectively. In each panel, the red circles and blue squares show data of individual female and male subjects, respectively. The red and blue lines with the light red and light blue bands show linear regressions in female and male subjects with their 95% confidence intervals, respectively. The light violet band shows the overlap between the confidence intervals of the regressions in female and male subjects.

Figure 1 shows the relationship between the mean NN interval and SDNN values. The linear slopes shown in the figure were all statistically significant, and the visual superimposition of the three panels of the figure shows that the slopes were a little dependent on the postural position, although, as expected, the mean NN intervals were gradually shortening during the supine → sitting → standing position changes. In the supine, sitting, and standing positions, the slopes in the female sub-population were 0.295, 0.242, and 0.288, respectively. The corresponding slopes in the male population were 0.470, 0.382, and 0.390, respectively. Very similar observations were also made when considering linear slopes between mean heart rate and SDHR values (Supplementary Figure S2).

**Figure 2:**
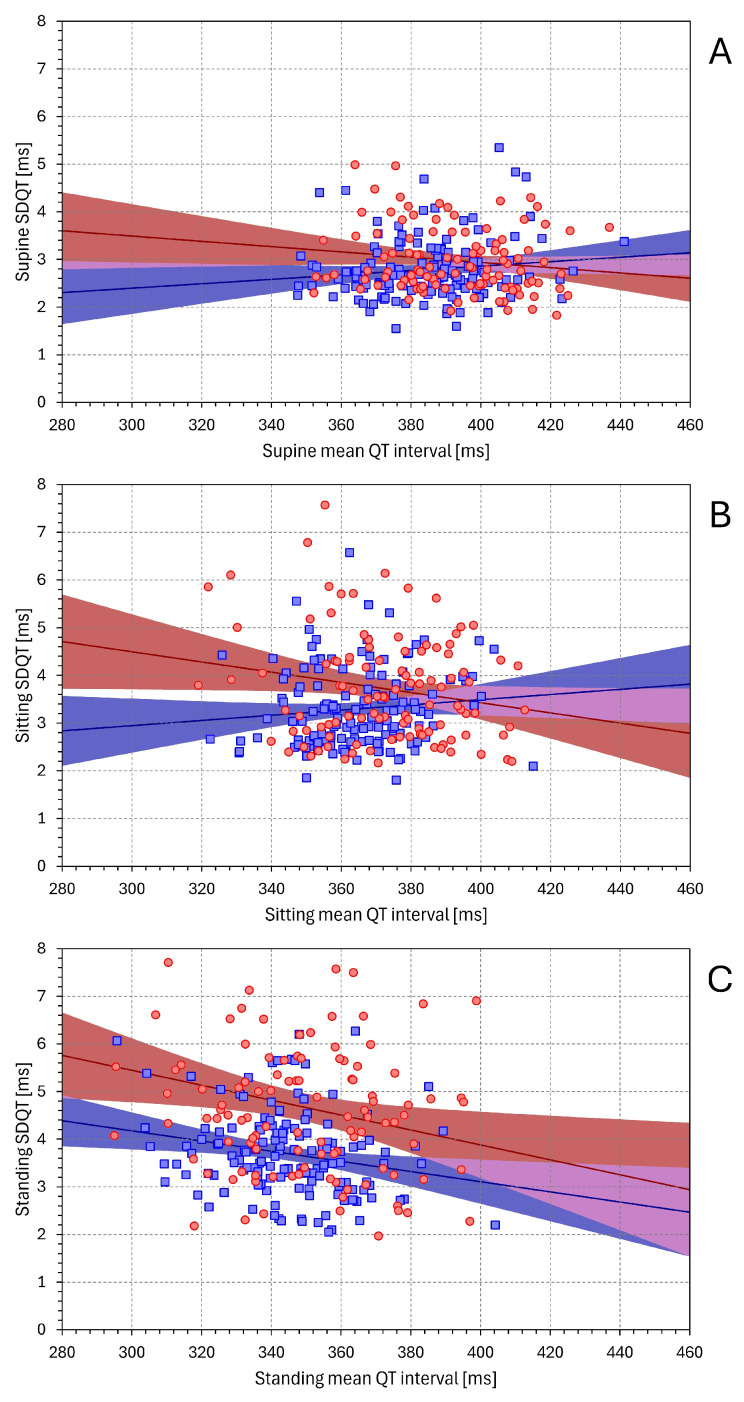
Relationship between mean QT interval duration and SDQT values. The layout of the figure and the meaning of the symbols are the same as in Figure 1.

On the contrary, as seen in Figure 2, the relationship between the mean QT interval duration and SDQT was not well defined. During the supine and sitting positions, the slopes of the SDQT/(mean QT) regression were not significantly different from zero. In the standing position, the slopes of this regression (−0.235 and −0.215 in females and males, respectively) were statistically significantly negative.

### 3.4. Postural Changes in SDNN and SDQT Values

**Figure 3:**
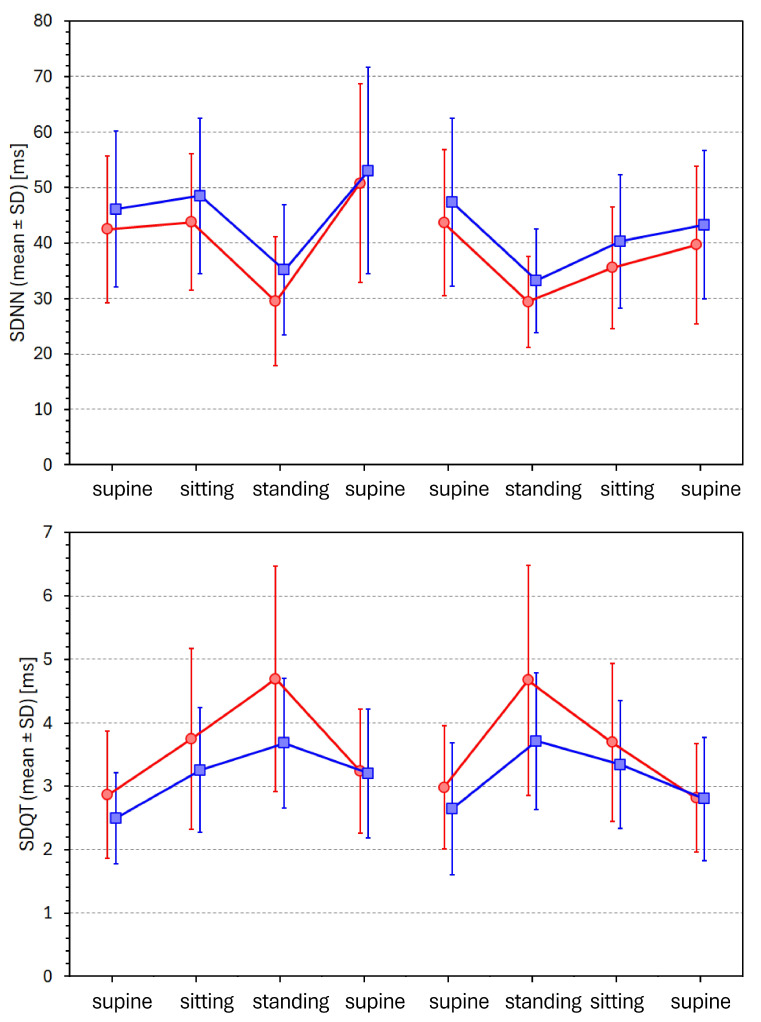
Development of SDNN values (top panel) and of SDQT values (bottom panel) during individual positions of separate tests performed in 4 drug-free investigation days (positions are listed below the panels). The red circles connected by red lines and the blue squares connected by blue lines are population means in female and male subjects. The error bars represent standard deviations in corresponding populations.

Figure 3 shows the development of SDNN and SDQT values during the individual postural positions. The differences that can be seen have also been confirmed when analysing data averaged over position categories. During supine, sitting, and standing positions, the SDNN values in females were 44.2 ± 13.5 ms, 39.7 ± 10.9 ms, and 29.4 ± 9.3 ms, respectively (all differences *p* < 0.0001). The corresponding values in males were 47.4 ± 13.9 ms, 44.4 ± 12.5 ms, and 34.2 ± 10 ms (all differences *p* < 0.0001). The sex comparison of SDNN values was not statistically significant in supine positions but was significant in sitting (*p* = 0.001) and standing (*p* = 0.0001) positions.

On the contrary, during supine, sitting, and standing positions, the SDQT values in females were 2.97 ± 0.69 ms, 3.72 ± 1.13 ms, and 4.68 ± 1.59 ms, respectively (all differences < 0.0001). The corresponding values in males were 2.79 ± 0.65 ms, 3.30 ± 0.83 ms, and 3.70 ± 0.90 ms, respectively (all differences *p* < 0.0001). The sex comparison of SDQT values was not statistically significant in the supine position but was significant in sitting (*p* = 0.001) and standing (*p* < 0.0001) positions.

Thus, while during the position transitions supine → sitting → standing, the mean SDNN values were decreasing with values lower in females compared to males, the situation with SDQT values was the exact opposite. During the same position transitions, the SDQT values were increasing with higher values in females compared to males.

Supplementary Figure S3 shows the regression relationship between SDQT and SDNN. In females, the data of the supine, sitting, and standing positions showed the slopes of 0.282 (*p* = 0.003), 0.201 (*p* = 0.037), and 0.196 (*p* = 0.042), respectively. The corresponding slope values in males were 0.419 (*p* < 0.001), 0.357 (*p* < 0.001), and 0.226 (*p* = 0.007). As seen in the figure, the mean square regression residuals were increasing when the positions were changed from supine → sitting → standing. In females, these residuals were 0.439 ms, 1.228 ms, and 2.461 ms. In males, the corresponding values were smaller while also increasing: 0.346 ms, 0.609 ms, 0.766 ms.

### 3.5. Postural Changes of “Mean Normalised” SDNN and SDQT Values

**Figure 4:**
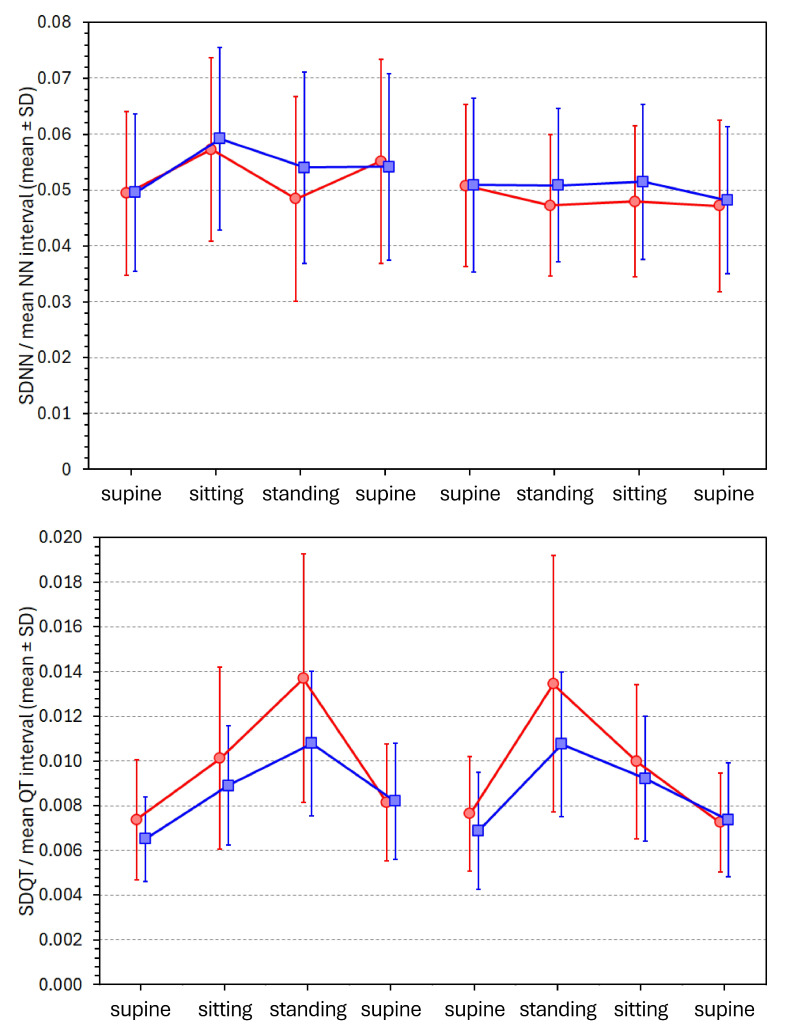
Development of proportions SDNN/(mean NN) (in the top panel) and of proportions SDQT/(mean QT) (in the bottom panel) during individual postural positions. The layout of the graphs and the meaning of the symbols are the same as in Figure 3.

Figure 4 shows the development of proportions SDNN/(mean NN) and SDQT/(mean QT) values during the individual postural positions.

Whilst the statistical comparison of these mean normalised SDNN and SDQT values was very similar to that of non-normalised SDNN and SDQT values, the effect of normalisation was very different. During supine, sitting, and standing positions, the SDNN/(mean NN) values in females were 0.051 ± 0.015, 0.053 ± 0.014, and 0.048 ± 0.015, respectively; the corresponding values in males were 0.051 ± 0.013, 0.055 ± 0.014, and 0.052 ± 0.015, respectively. The corresponding values of normalised SDQT/(mean QT) values in females were 0.0076 ± 0.0019, 0.0101 ± 0.0032, and 0.0136 ± 0.0051, respectively; the corresponding values in males were 0.0072 ± 0.0017, 0.0091 ± 0.0023, and 0.0108 ± 0.0028, respectively.

Thus, while the normalisation for mean NN interval led to lower inter-position and sex differences for SDNN, the inter-position and sex differences in SDQT were enhanced by normalisation for the mean QT interval.

Supplementary Figure S4 shows the regression relationship between the normalised SDNN/(mean NN) and SDQT/(mean QT) values. Compared to the regressions between the non-normalised values (as shown in Supplementary Figure S3), the slopes between the normalised values were steeper. In females, the slopes corresponding to the supine, sitting, and standing positions were 0.381 (*p* < 0.001), 0.302 (*p* = 0.002), and 0.305 (*p* = 0.001), respectively. In males, the corresponding slope values were 0.427, 0.364, and 0.360 (all *p* < 0.001).

Comparison of Supplementary Figures S3 and S4 also shows that while prior to normalisation, the slopes were increasing with position changes supine → sitting → standing, the slopes were decreasing after normalisation (the difference is particularly noticeable during the supine → sitting position change).

### 3.6. Postural Changes in QT Variability Index

**Figure 5:**
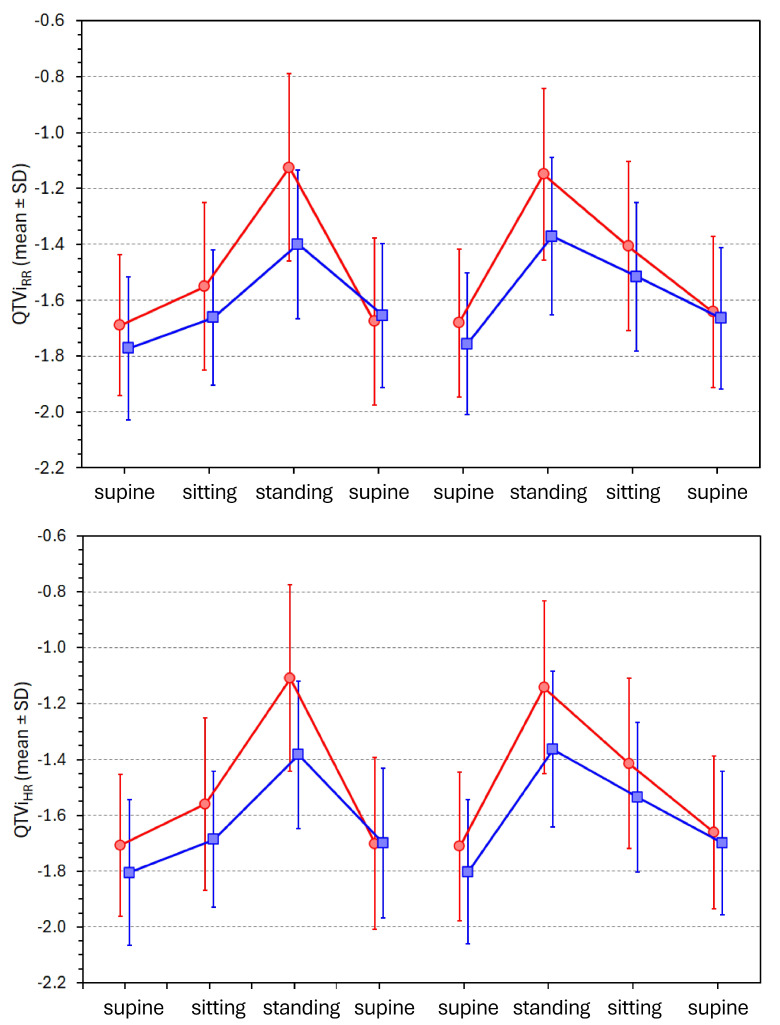
Development of QTVi_RR_ values (top panel) and of QTViHR values (bottom panel) during individual postural positions. The layout of the graphs and meaning of the symbols are the same as in Figure 3.

Figure 5 shows the development of QTVi values during individual postural positions. As seen in the Figure, the changes due to the postural position changes are substantial. In averaged data per position, the differences between supine, sitting, and standing were all highly significant in both females and males (all *p* < 0.0001). The sex differences were not significant in the supine position but were significant in sitting (*p* < 0.001) and standing (*p* < 0.0001) positions. These observations applied equally to both QTVi_RR_ and QTVi_HR_.

**Figure 6:**
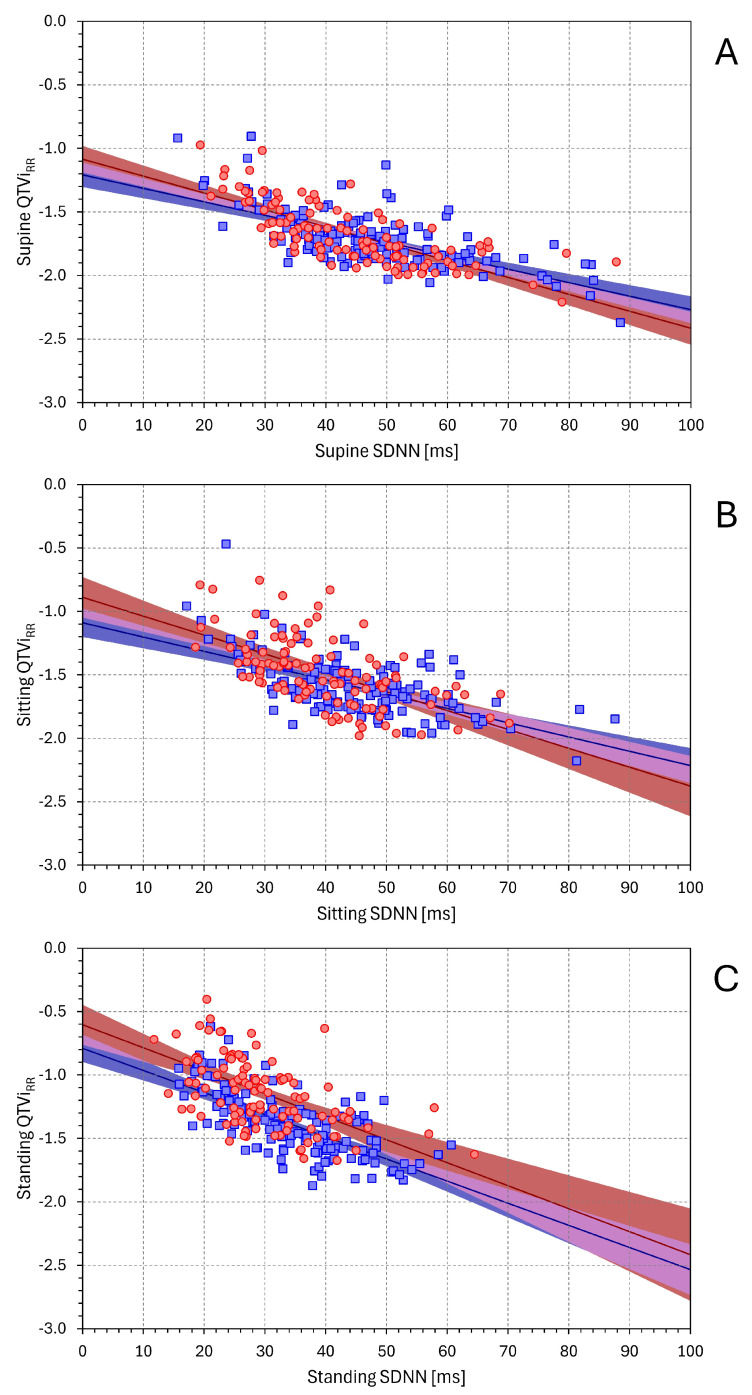
Relationship between QTVi_RR_ and SDNN values. The layout of the figure and the meaning of the symbols are the same as in Figure 1.

**Figure 7:**
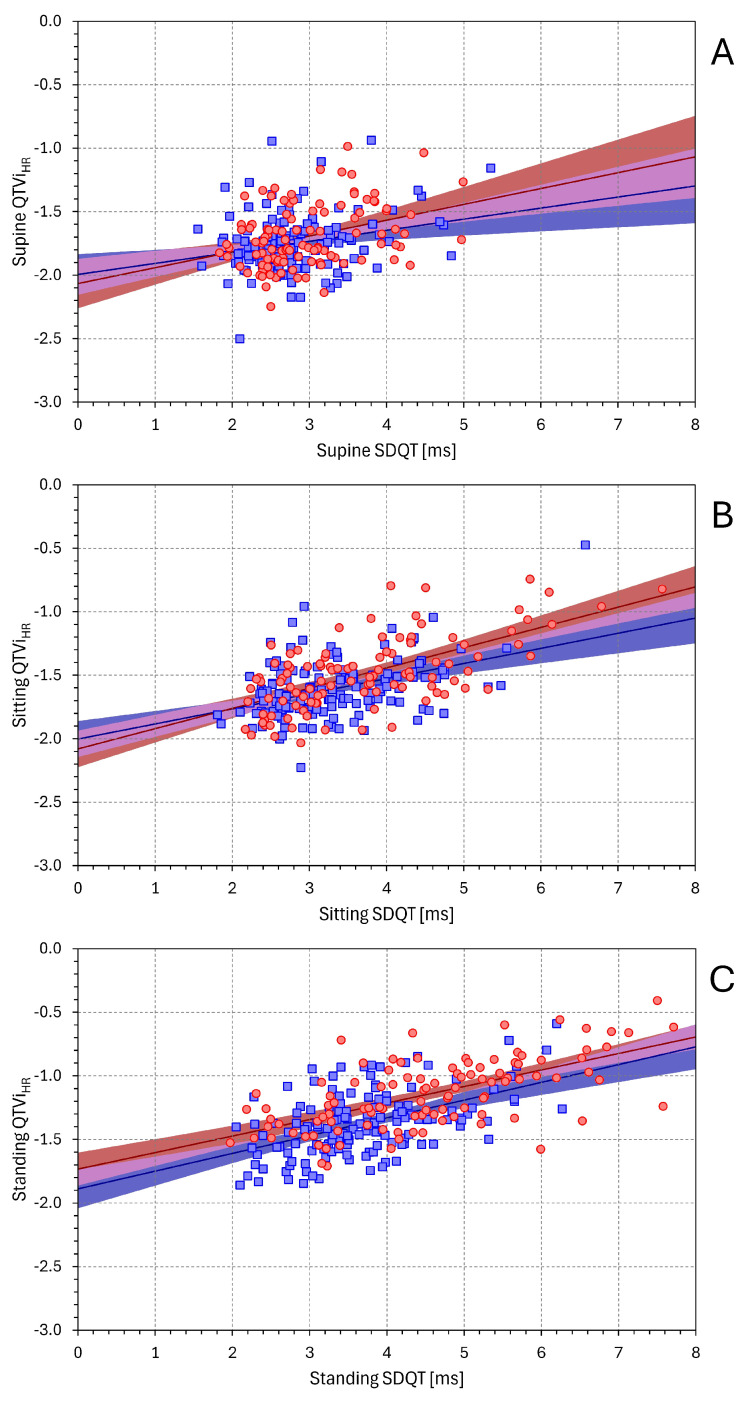
Relationship between QTViRR and SDQT values. The layout of the figure and the meaning of the symbols are the same as in Figure 1.

Figure 6 and Figure 7 show the linear regressions corresponding to the dependencies of QTVi_RR_ on SDNN and on SDQT. Supplementary Figures S5 and S6 show the same analyses for QTVi_HR_.

All slopes between QTVi_RR_ on SDNN and between QTVi_RR_ on SDQT were statistically significant (*p* < 0.001 for all). The strength of the dependency of QTVi_RR_ on SDNN and on SDQT was not systematically different in females (t-statistics of −11.6 vs. 3.9, −7.5 vs. 8.3, and −6.9 vs. 9.1 for supine, sitting, and standing positions, respectively). In males, however, QTVi_RR_ depended more strongly on SDNN than on SDQT (t-statistics of −10.7 vs. 3.7, −9.1 vs. 6.2, and −11.4 vs. 6.5 for supine, sitting, and standing positions, respectively). The dependency of QTVi_HR_ on SDNN and on SDQT showed practically the same results.

**Figure 8:**
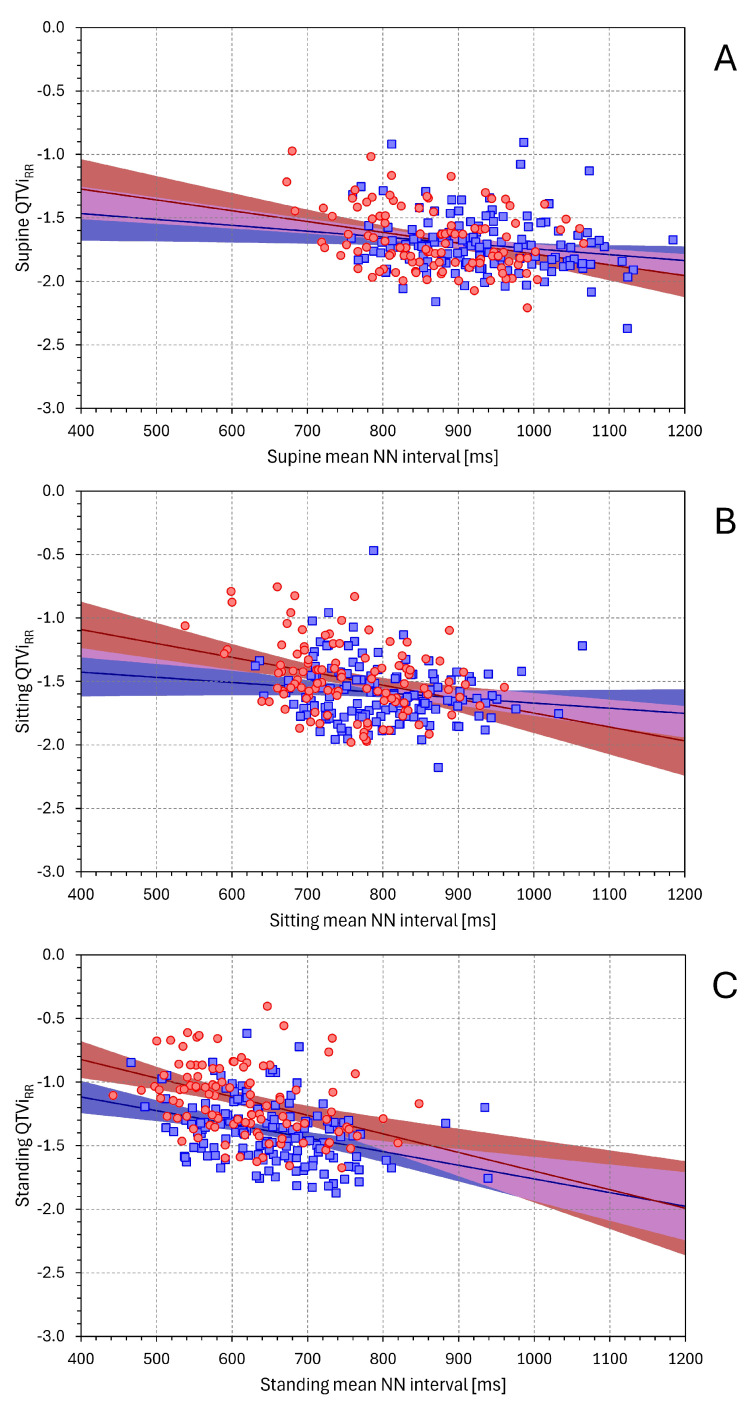
Relationship between QTViRR and mean NN interval values. The layout of the figure and the meaning of the symbols are the same as in Figure 1.

Figure 8 shows the relationship between the QTVi_RR_ and mean NN interval values. Although the influence of mean NN interval values was weaker than that of SDNN and SDQT, it systematically reached statistical significance in females (*p* < 0.001 for all three postural positions). In males, the statistical significance was noted only in supine and standing positions (*p* = 0.023 and *p* < 0.001, respectively). Again, the relationship between the QTVi_HR_ and mean NN interval values was practically the same.

These observations were consistent with the results of multivariable regression analyses relating QTVi to a linear combination of mean NN, mean QT, SDNN, and SDQT. Generally, the mean QT interval duration did not significantly contribute to QTVi_RR_ apart from the analyses in males in supine (*p* = 0.012) and standing positions (*p* = 0.002). The linear contributions of mean RR, SDNN, and SDQT to QTVi_RR_ were all statistically significant (all *p* < 0.001). In terms of the characteristics of the regression models, the *t*-values were systematically substantially smaller for mean RR in comparison to those for SDNN and SDQT. The *t*-values for SDNN were similar to those for SDQT in females (−31.3 vs. 22.0 in supine positions, −27.8 vs. 29.0 in sitting position, and −26.9 vs. 30.0 in standing position) but were systematically stronger in males (−30.1 vs. 22.6 in supine position, −33.2 vs. 30.0 in sitting position, and -37.3 vs. 31.0 in standing position). Thus, QTVi_RR_ was similarly dependent on SDNN and SDQT in females but more strongly dependent on SDNN than on SDQT in males.

The results for the QTVi_HR_ analyses were the same (although with tiny differences in numerical values).

### 3.7. Influence of Age

**Figure 9:**
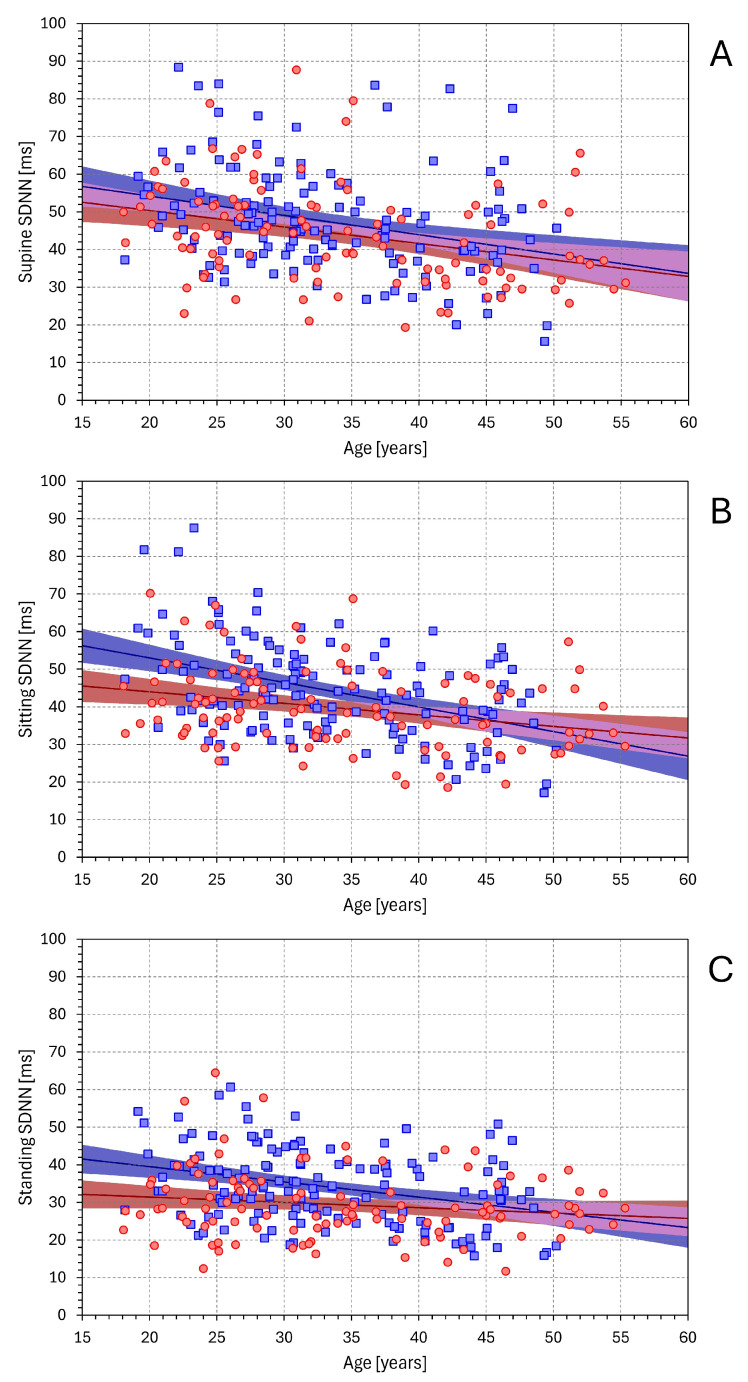
Relationship between SDNN values and the age of study subjects. The layout of the figure and the meaning of the symbols are the same as in Figure 1.

Figure 9 shows the dependency of SDNN on age. As expected, independent of the postural position, SDNN decreased with age. The decrease was statistically significant in males in all positions (*p* < 0.001 for all), but in females, the decrease was statistically significant only in supine (*p* < 0.001) and sitting (*p* = 0.003) positions.

**Figure 10:**
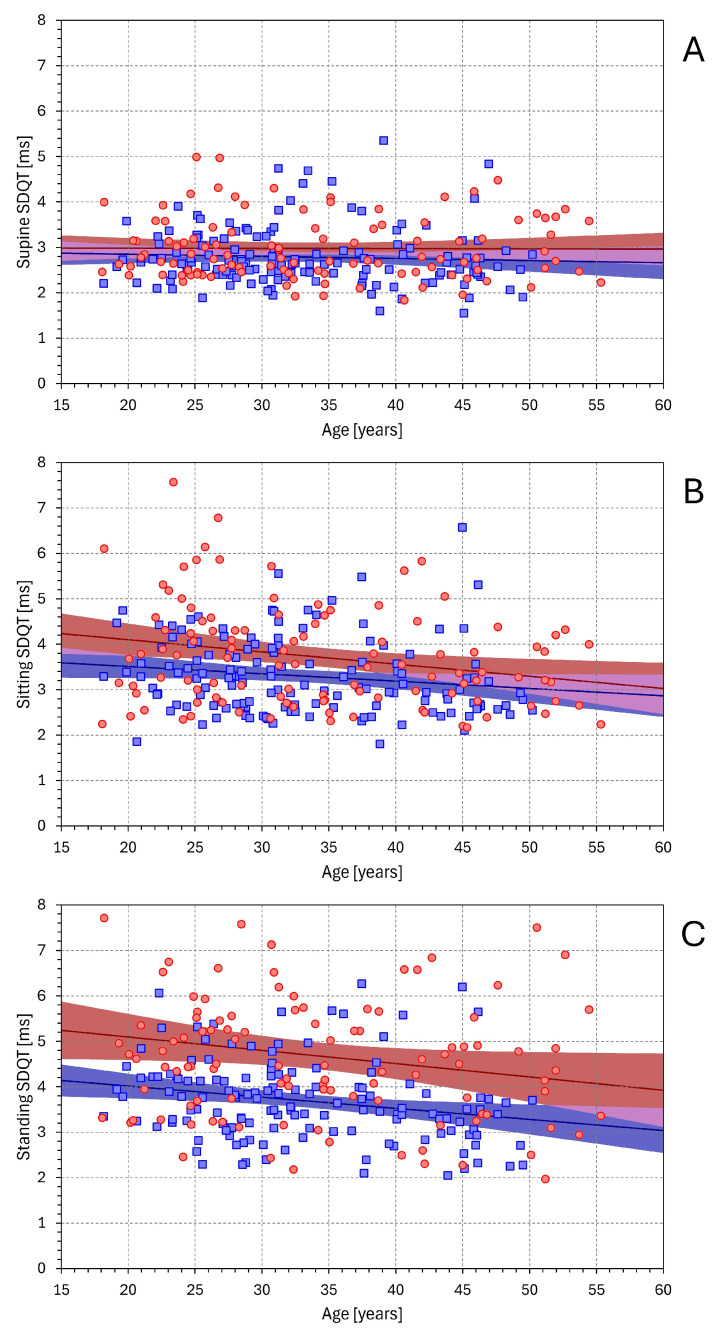
Relationship between SDQT values and the age of study subjects. The layout of the figure and the meaning of the symbols are the same as in Figure 1.

Figure 10 shows the corresponding dependency of SDQT on age. Again, independent of the postural positions, SDQT decreased with age. This decrease showed only non-significant trends in the supine position but was statistically significant in sitting (*p* = 0.01 in females and *p* = 0.05 in males) and in the standing position (*p* = 0.05 in females and *p* = 0.007 in males).

**Figure 11:**
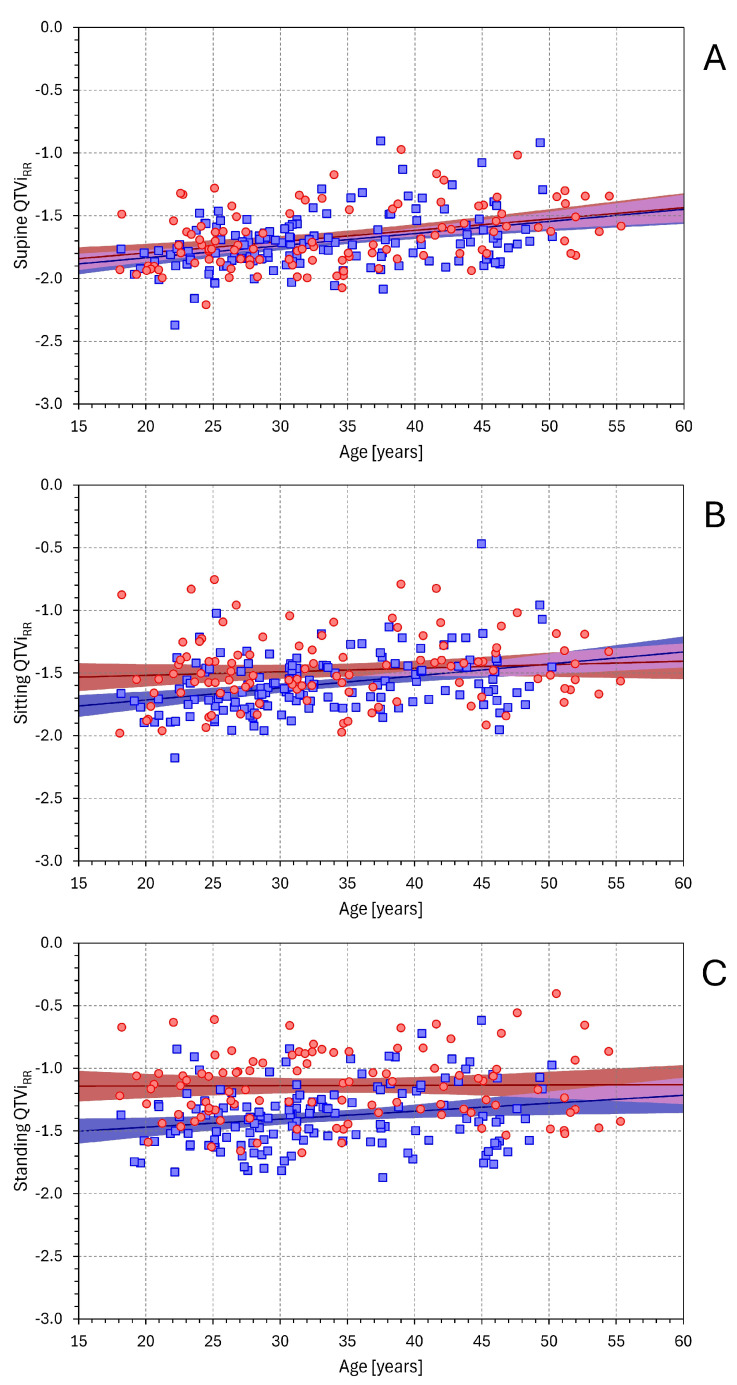
Relationship between QTVi_RR_ values and the age of study subjects. The layout of the figure and the meaning of the symbols are the same as in Figure 1.

The dependency of QTVi_RR_ on age is shown in Figure 11. In all postural positions, an increase in QTVi_RR_ with age was observed. This was not statistically significant in females in sitting and standing positions but was significant in females in the supine position (*p* < 0.001) and in males in all positions (*p* < 0.001, *p* < 0.001, and *p* = 0.014 in supine, sitting, and standing positions, respectively). When analysing the dependency of QTVi_HR_ on age (Supplementary Figure S7), the results were practically the same.

## 4. Discussion

The study provides insight into the relationship between indices that are combined in the QTVi. The observations that we have made include technical, physiological, and clinical aspects related to the risk prediction by QTVi.

As published many times in the past, we have observed that SDNN values are positively correlated with the mean duration of NN intervals [36,37]. Heart rate increases (i.e., mean NN interval decreases) during the postural changes supine → sitting → standing have also been described previously [27]. As seen in Figure 1 (and similarly in Supplementary Figure S2), these decreases in mean NN intervals occurred together with decreases in SDNN values. Naturally, the mean NN interval decreases during the postural changes, which were accompanied by decreases in the mean QT interval duration. However, contrary to the SDNN decreases, SDQT values appeared to increase (compare individual panels of Figure 2), albeit not systematically with mean QT interval durations. Thus, whilst it makes physiologic sense to normalise SDNN for mean NN interval durations, similar normalisation of SDQT for mean QT interval duration lacks a physiologic background.

The calculation of SDNN/(mean NN) indices is naturally a very approximate and limited normalisation of the SDNN values. Other and more technically advanced models of the HRV/heart-rate relationship have been proposed [38]. Nevertheless, a comparison of the top panels of Figure 3 and Figure 4 confirms that relating the SDNN values to mean NN interval durations suppresses the variability of non-normalised SDNN values. On the contrary, a comparison of the bottom panels of Figure 3 and Figure 4 shows that rather than normalising the SDQT values, their simple division by mean QT interval durations enhances the differences between postural positions. Indeed, when SDQT values increase with decreasing mean QT interval (see panel C of Figure 2), the relationship SDQT/(mean QT) is not only without physiologic justification but also technically misplaced.

As we have already shown in the section on the QTVi definition, the combination of mean NN and mean QT intervals as correction factors in the QTVi formula leads to a somewhat strange element [(mean NN)/(mean QT)], the logarithm of which is added to the logarithm of (SDQT/SDNN). It is therefore not surprising that the QTVi values show an inverse relationship to the mean NN interval (and thus a positive relationship to the underlying heart rate). When the mean NN interval decreases, so does the mean QT interval. However, as is known from a number of QTc rate correction formulas, the proportion between the NN interval and QT interval changes is skewed towards the NN intervals. The same bias of the changes in the SDQT/SDNN proportion towards the SDNN changes is documented in the slope values of the relationship between the SDQT and SDNN values (see the statistical observations that we have made with the graphs in Supplementary Figure S3).

In addition to these technical complexities, the composite nature of the QTVi formula also appears to restrict its use in physiological investigations. The name *QT variability index* might create an impression that it mainly reflects the beat-to-beat variability of QT interval durations. While it is true that QTVi is closely related to SDQT (see Figure 7), it is also, and perhaps more tightly, albeit negatively, related to SDNN (see Figure 6). This is likely one of the reasons why QTVi values reported in different studies [25] are more variable than those available in the literature. Hence, establishing a reference distinction between normal and abnormal values is problematic.

The relationship to age that we have investigated also shows another facet of the problem with physiological interpretation. While we observed that SDQT values tended to decrease with increasing age, the opposite was true for QTVi values. This example clearly shows the possibility of erroneous and/or unfounded physiological conclusions.

The seminal publication that defined QTVi showed its applicability in the prediction of adverse events. A number of subsequent studies reproduced the findings. Nevertheless, regrettably, systematic studies are lacking that would investigate the predictive power of QTVi in comparison to that of HRV and of mean heart rate in multivariable Cox regression models. Both decreased HRV [9,17,39] and increased heart rate [40] are well-known risk factors that have been well established to predict adverse outcomes not only in cardiac patients but also in a multitude of other clinical conditions, including unselected general populations. It can therefore be questioned whether the risk prediction reported by increased QTVi was driven by increased SDQT or whether it was more (or perhaps exclusively) driven by decreased HRV combined with increased heart rate.

Although the QTVi formula also includes the averaged QT interval duration, our multivariable regression analyses have not found any systematically significant influence of QT interval duration on the QTVi values. The same was true when we changed the uncorrected QT interval duration for Fridericia corrected QTc intervals (details not shown).

The publication that introduced QTVi did not explain the reasoning for the rather complex formula of the index. It might perhaps be assumed that originally, the authors expected that (a) the mean QT interval durations would influence the QT interval variability in the same way as the mean RR interval influences the RR interval variability, and (b) the QT interval durations would respond to the duration of the preceding RR interval, which would lead to the proportion of SDQT/SDNN expressing only QT interval variability independent of the RR interval influence. The assumption (a) is clearly contradicted by our Figure 1 and Figure 2. The assumption (b) is contradicted by the so-called QT/RR hysteresis, which shows that QT interval duration does not respond to individual RR interval changes but gradually responds to the changes in underlying heart rate over a number of preceding RR intervals.

It might be supposed that the logarithmic transformation was introduced into the QTVi formula [26] to make the resulting values suitable for assessment by parametric statistical tests. Nevertheless, as we have shown in the section on QTVi definition, the logarithmic transformation leads to a somewhat problematic combination of initial elementary measurements. Our multivariable regression analysis suggests that SDQT and SDNN are, despite their limited correlation (see Supplementary Figure S3), the dominant components of QTVi. The simplified logarithmic form QTVi ~log(SDQT)−log(SDNN) shows that for purely mathematical reasons, QTVi increases when SDQT increases and/or SDNN decreases, while the QTVi formula cannot distinguish between these two sources of change. This makes the interpretation of QTVi changes less clear and challenges the usual presumption that QTVi is a direct measure of cardiac repolarisation instability.

Our concerns with the QTVi definition are purely related to the physiological interpretation of the measurements. We are certainly not trying to dispute all the previous known and well-reviewed [25] studies that found the QTVi increases are indicative of future adverse outcomes. Undisputably, increased beat-to-beat variability of T wave morphology and/or of QT interval duration is indicative of repolarisation abnormalities with all well-known clinical implications [21,25]. We merely point out that the QTVi definition incorporates both QT interval variability and HRV and thus cannot distinguish between the clinical implications of repolarisation abnormalities that lead to the increases in QT variability and of autonomic abnormalities that lead to HRV decreases and heart rate increases. We are therefore suggesting that in future studies that use QTVi, the HRV indices and underlying heart rate values need to be included among measured indices so that multivariable regression methods might be used to distinguish between the possible sources of QTVi changes and/or observed population differences.

###  Limitations 

Naturally, our observations also suffer from several limitations. The most important restriction of our data stems from the use of data and ECG recordings of healthy subjects. As previously stated, the subjects also underwent strict enrolment in a clinical pharmacology study. Moreover, our selection of stable heart rate segments might have simplified the SDQT/SDNN relationship. We are thus unable to comment on whether the same interpretative restrictions of QTVi would also apply to clinical populations such as patients with ischaemic heart disease. Nevertheless, any parameter that is not sufficiently stable in a healthy population might be expected to suffer from similar problems also when derived from clinical recordings. In particular, the analysis of the healthy population does not allow us to investigate the predictive strength of QTVi. In ischaemic heart disease patients and other clinical populations, depressed HRV and increased heart rate are well-known risk factors, and thus, our observations would likely apply. We have also investigated the relationship between different QTVi components using population data. Similar to the subject-specific QT/RR profiles [41], it might be expected that the QTVi/SDNN, QTVi/SDQT, and other similar profiles would also show differences between different individuals. While our data would allow such an analysis, the topic is well beyond the scope of this study and needs to be addressed in a separate investigation. Finally, while we excluded subjects with missing data and/or technically unsuitable ECG recordings, we have not assessed the noise in the analysed ECG sections. It has previously been reported that QT interval variability is highly influenced by ECG signal quality [42]. The dependence of our observations on the underlying ECG noise also needs to be addressed in a separate investigation.

## 5. Conclusions

Despite these limitations, our analyses allow us to conclude that the definition of the QT variability index determines the results, influenced by factors that are not related to the beat-to-beat changes in the QT interval durations. It can be strongly recommended that in future studies, simpler measurements of the QT interval variability are used together with HRV and heart rate factors. Multivariable statistical models need to be used to eliminate the possibility that findings of QT interval variability are not predominantly based on beat-to-beat variabilities of other ECG components.

## Figures and Tables

**Table 1:** Measured ECG data. QTcF—Fridericia corrected QT interval; SSMD—strictly standardised mean differences. See the text for all other abbreviations.

Parameter	Position	Females	Males	*p*-Value	SSMD
Mean NN interval [ms]	Supine	868.8 ± 88.2	932.2 ± 90.5	7.05 × 10^−8^	−0.5011
	Sitting	753.7 ± 82.2	800.3 ± 81.2	1.21 × 10^−5^	−0.4037
	Standing	615.0 ± 83.4	650.0 ± 81.2	9.99 × 10^−4^	−0.3011
Mean heart rate [bpm]	Supine	70.33 ± 7.27	65.46 ± 6.30	8.23 × 10^−8^	0.5059
	Sitting	81.16 ± 9.06	76.33 ± 7.56	1.25 × 10^−5^	0.4087
	Standing	100.01 ± 13.57	94.46 ± 11.66	7.82 × 10^−4^	0.3105
SDNN [ms]	Supine	44.15 ± 13.55	47.45 ± 13.87	6.03 × 10^−2^	−0.1699
	Sitting	39.65 ± 10.94	44.42 ± 12.49	1.48 × 10^−3^	−0.2871
	Standing	29.42 ± 9.27	34.18 ± 10.04	1.31 × 10^−4^	−0.3486
SDHR [bpm]	Supine	3.675 ± 1.107	3.517 ± 0.978	2.39 × 10^−1^	0.1075
	Sitting	4.317 ± 1.268	4.322 ± 1.179	9.75 × 10^−1^	−0.0028
	Standing	4.661 ± 1.460	4.808 ± 1.404	4.22 × 10^−1^	−0.0728
Mean QT interval [ms]	Supine	392.5 ± 18.3	385.2 ± 17.1	1.43 × 10^−3^	0.2925
	Sitting	372.6 ± 20.2	364.6 ± 16.1	8.63 × 10^−4^	0.3097
	Standing	348.9 ± 23.9	345.0 ± 18.1	1.65 × 10^−1^	0.1279
Mean QTcF interval [ms]	Supine	400.7 ± 14.6	389.3 ± 13.5	1.09 × 10^−9^	0.5770
	Sitting	388.3 ± 16.3	376.7 ± 12.7	5.10 × 10^−9^	0.5608
	Standing	374.4 ± 19.5	367.4 ± 14.1	1.82 × 10^−3^	0.2915
SDQT [ms]	Supine	2.975 ± 0.687	2.788 ± 0.646	3.00 × 10^−2^	0.1978
	Sitting	3.720 ± 1.126	3.299 ± 0.832	1.27 × 10^−3^	0.3009
	Standing	4.682 ± 1.592	3.695 ± 0.895	3.78 × 10^−8^	0.5401
QTVi_RR_	Supine	−1.672 ± 0.241	−1.712 ± 0.219	1.84 × 10^−1^	0.1209
	Sitting	−1.478 ± 0.276	−1.589 ± 0.231	8.99 × 10^−4^	0.3075
	Standing	−1.137 ± 0.300	−1.386 ± 0.253	4.61 × 10^−11^	0.6341
QTVi_HR_	Supine	−1.695 ± 0.246	−1.752 ± 0.226	6.27 × 10^−2^	0.1696
	Sitting	−1.487 ± 0.283	−1.610 ± 0.232	3.00 × 10^−4^	0.3362
	Standing	−1.125 ± 0.301	−1.373 ± 0.251	5.24 × 10^−11^	0.6327

## Data Availability

The data supporting the conclusions of this article will be made available by the authors pending the approval by the sponsors of the original pharmaceutical studies. Requests for the data are to be sent to the corresponding author together with a detailed plan of the proposed analyses.

## Supplementary Materials

The online supporting figures can be downloaded at: https://www.mdpi.com/article/10.3390/diagnostics16030502/s1. Figure S1: Relationship between average of QTViHR and QTViHR and the difference QTViRR minus QTViHR in supine, sitting, and standing positions are shown in panels A, B, and C, respectively. Figure S2: Relationship between heart rate and SDHR values in supine, sitting, and standing positions are shown in panels A, B, and C, respectively. Figure S3: Relationship between SDNN and SDQT values in supine, sitting, and standing positions are shown in panels A, B, and C, respectively. Figure S4: Relationship between (SDNN /mean NN) and (SDQT/mean QT) values in supine, sitting, and standing positions are shown in panels A, B, and C, respectively. Figure S5: Relationship between SDNN and QTVi_HR_ values in supine, sitting, and standing positions are shown in panels A, B, and C, respectively. Figure S6: Relationship between SDQT and QTVi_HR_ values in supine, sitting, and standing positions are shown in panels A, B, and C, respectively. Figure S7: Relationship between Age and QTVi_HR_ values in supine, sitting, and standing positions are shown in panels A, B, and C, respectively.

## Abbreviations

The following abbreviations are used in this manuscript:

ECG	Electrocardiogram
HRV	Heart rate variability
ms	Millisecond
NN	Normal-to-normal RR interval
QTcF	Fredericia corrected QT interval
QTV	QT interval variability
QTVi	QT interval variability index
QTViRR	Variant of QT interval variability index based on RR interval variance
QTViHR	Variant of QT interval variability index based on heart rate variance
SD	Standard deviation
SDHR	Standard deviation of heart rate values
SDNN	Standard deviation of normal-to-normal RR intervals
SDQT	Standard deviation of QT intervals
SSMD	Strictly standardised mean differences
