# Supplementary material for: Beat-to-Beat QT Variability: A Population Study of the QT Variability Index Composition
Source: Diagnostics (Basel). 2026 Feb 6;16(3):502. doi: 10.3390/diagnostics16030502 (PMC12896582; doi:10.3390/diagnostics16030502)
Supplement: Supplementary file 1 [file diagnostics-16-00502-s001.zip › diagnostics-4081147-supplementary.pdf]

diagnostics

MDPI

Supplementary Figures

Beat to beat QT variability: Population study of the QT variability index composition

Jan Řehoř [1,2], Kateřina Helánová [1,2,*], Martina Šišáková [1,2], Tomáš Novotný [1,2], Irena Andršová [1,2], Marek Malik [3,2]

[1] Department of Internal Medicine and Cardiology, University Hospital Brno, Jihlavská 20, 625 00 Brno, Czech Republic
[2] Department of Internal Medicine and Cardiology, Faculty of Medicine, Masaryk University, Jihlavská 20, 625 00 Brno, Czech Republic
[3] National Heart and Lung Institute, Imperial College, 72 Du Cane Rd, Shepherd's Bush, London W12 0NN, England
* Correspondence: helanova.katerina@fnbrno.cz

 https://doi.org/10.3390/diagnostics16030502

Supplementary Figure S1

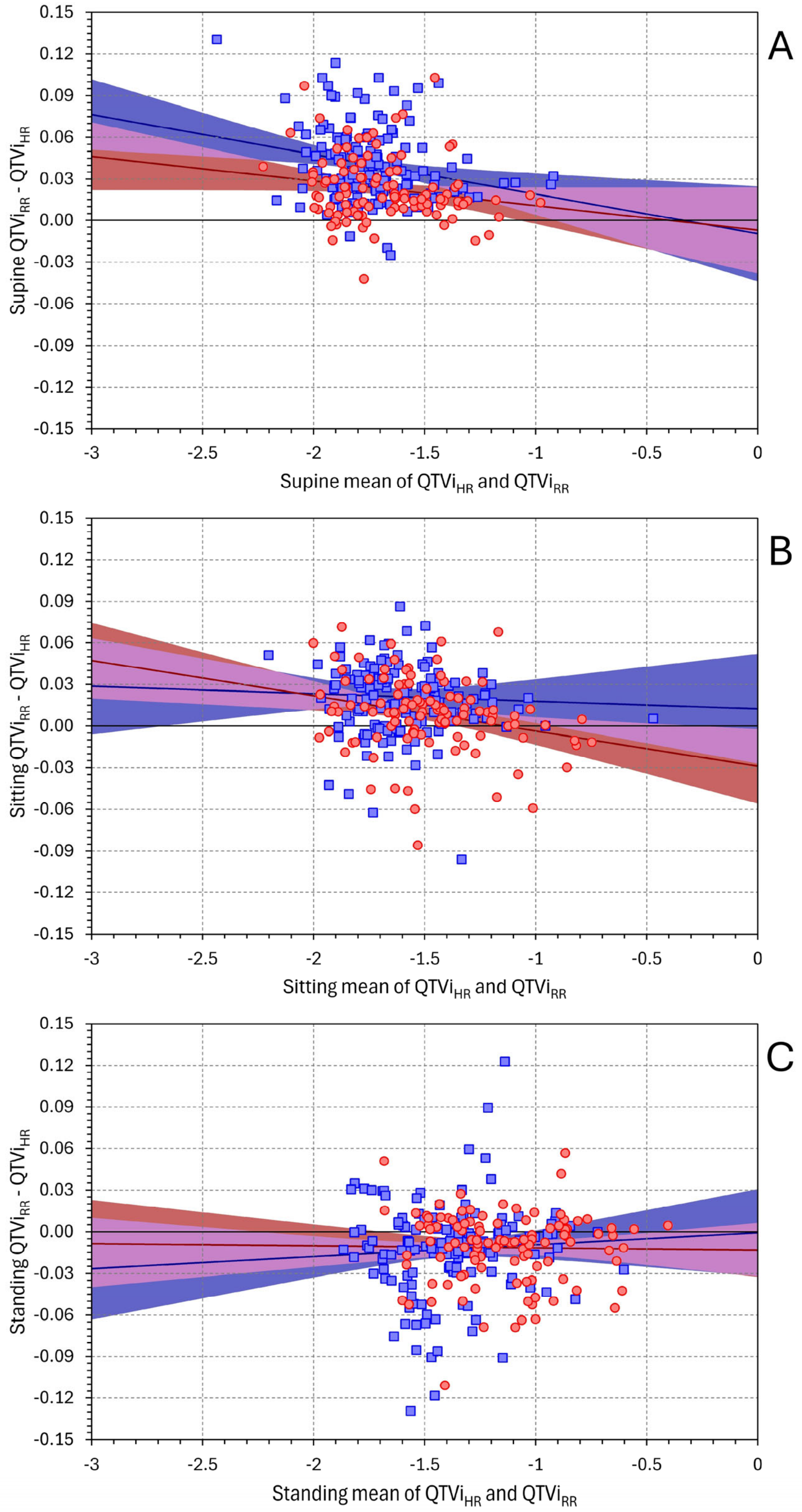

Figure S1: Relationship between average of $QTVi_{HR}$ and $QTVi_{HR}$ and the difference $QTVi_{RR}$ minus $QTVi_{HR}$ in supine, sitting, and standing positions are shown in panels A, B, and C, respectively. In each panel, the red circles and blue squares show data of individual female and male subjects, respectively. The red and blue lines with the light red and light blue bands show linear regressions in female and male subjects with their 95% confidence intervals, respectively. The light violet band shows the overlap between the confidence intervals of the regressions in female and male subjects.

https://doi.org/10.3390/diagnostics16030502

Supplementary Figure S2

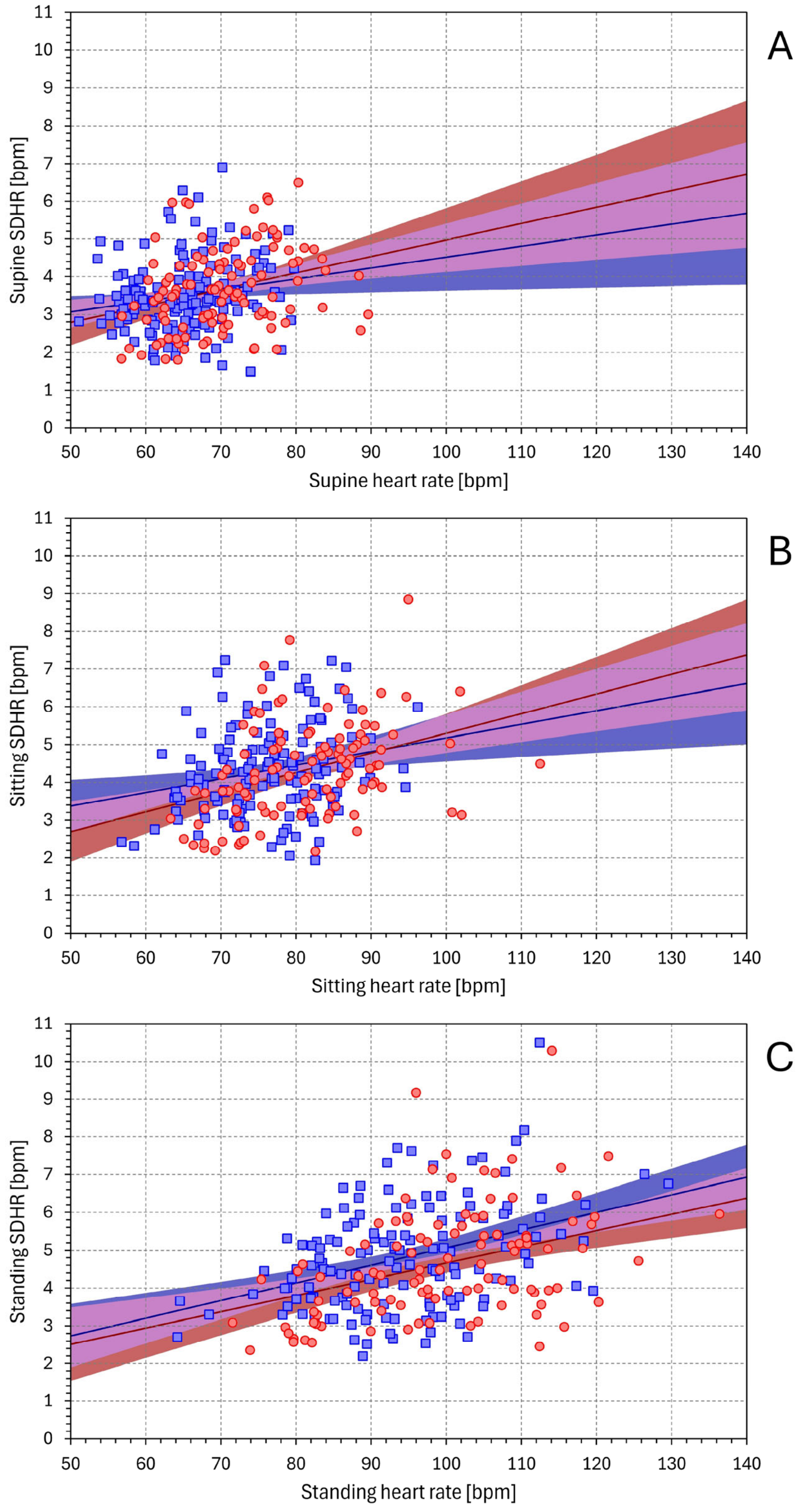

Figure S2: Relationship between heart rate and SDHR values in supine, sitting, and standing positions are shown in panels A, B, and C, respectively. In each panel, the red circles and blue squares show data of individual female and male subjects, respectively. The red and blue lines with the light red and light blue bands show linear regressions in female and male subjects with their 95% confidence intervals, respectively. The light violet band shows the overlap between the confidence intervals of the regressions in female and male subjects.

https://doi.org/10.3390/diagnostics16030502

Supplementary Figure S3

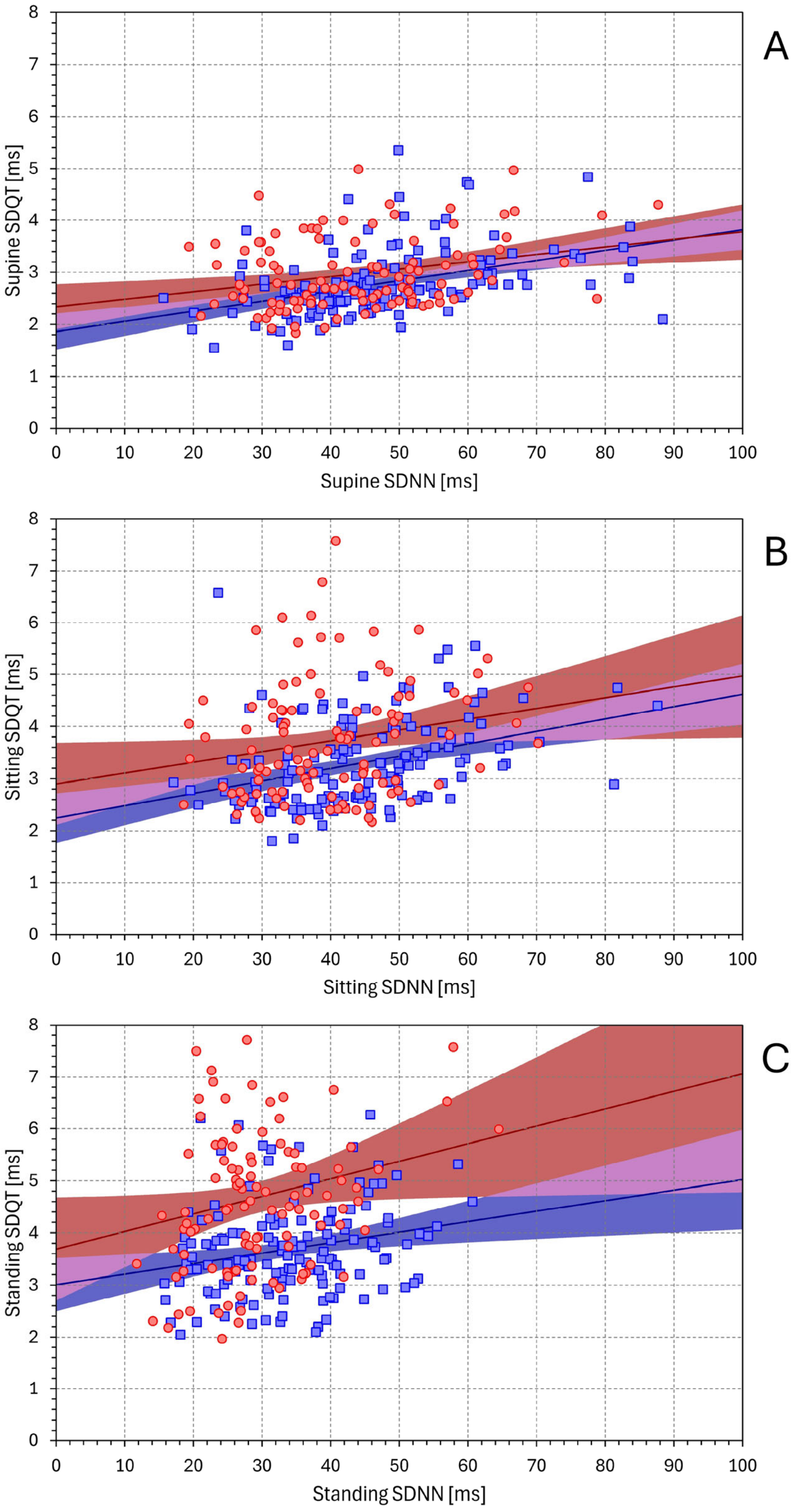

Figure S3: Relationship between SDNN and SDQT values in supine, sitting, and standing positions are shown in panels A, B, and C, respectively. In each panel, the red circles and blue squares show data of individual female and male subjects, respectively. The red and blue lines with the light red and light blue bands show linear regressions in female and male subjects with their 95% confidence intervals, respectively. The light violet band shows the overlap between the confidence intervals of the regressions in female and male subjects.

https://doi.org/10.3390/diagnostics16030502

Supplementary Figure S4

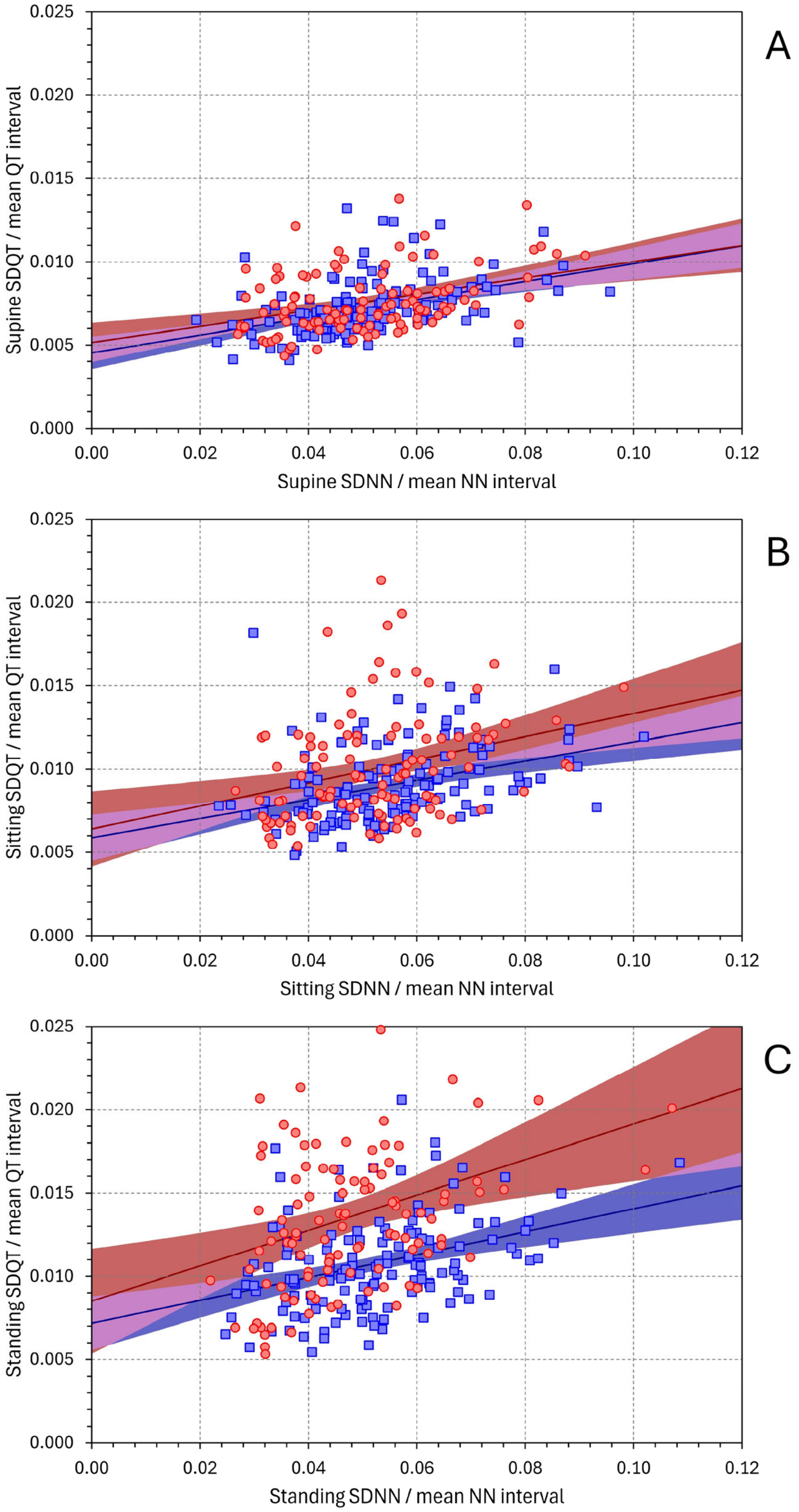

Figure S4: Relationship between (SDNN /mean NN) and (SDQT / mean QT) values in supine, sitting, and standing positions are shown in panels A, B, and C, respectively. In each panel, the red circles and blue squares show data of individual female and male subjects, respectively. The red and blue lines with the light red and light blue bands show linear regressions in female and male subjects with their 95% confidence intervals, respectively. The light violet band shows the overlap between the confidence intervals of the regressions in female and male subjects.

https://doi.org/10.3390/diagnostics16030502

Supplementary Figure S5

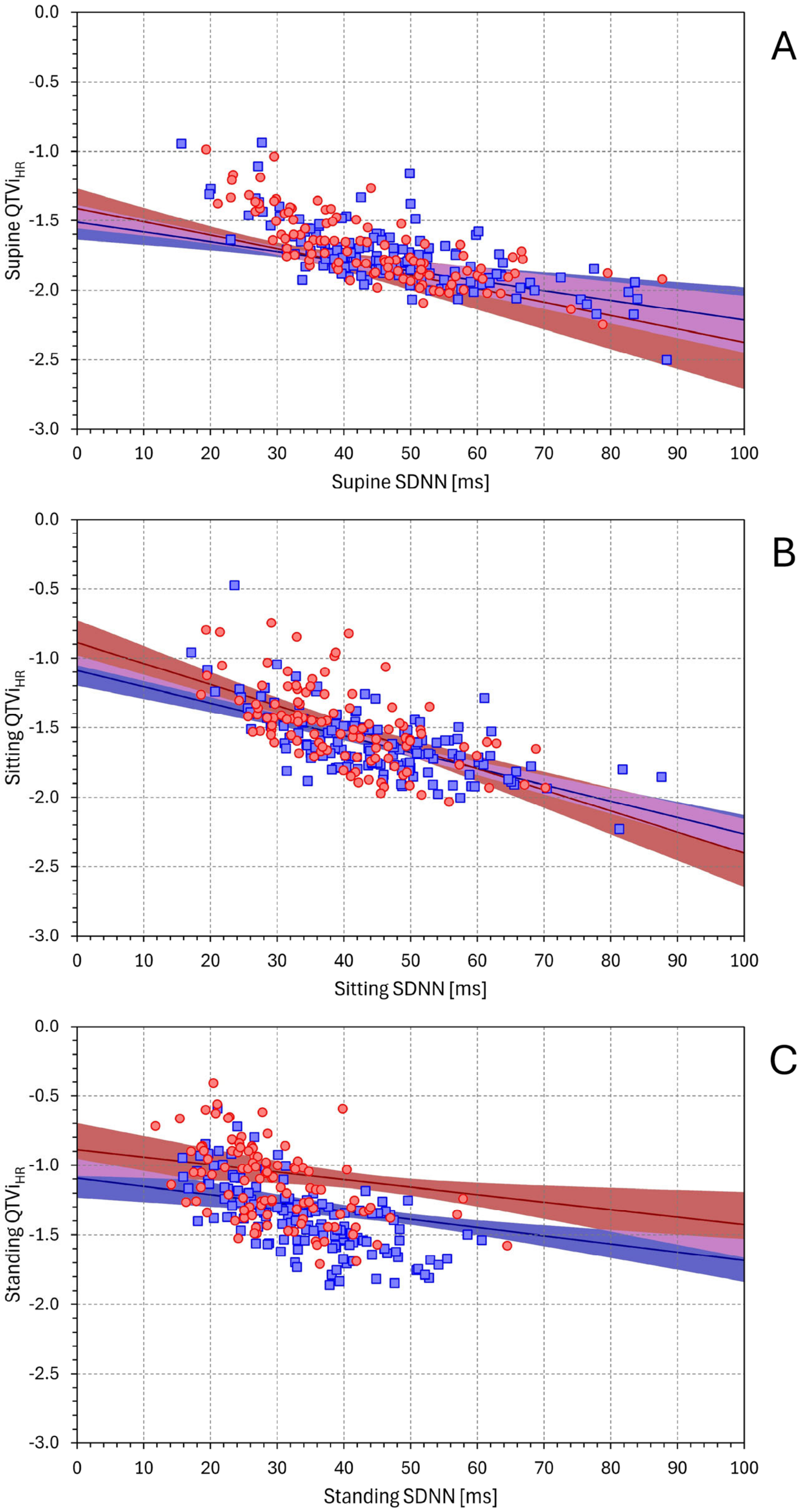

Figure S5: Relationship between SDNN and $QTVi_{HR}$ values in supine, sitting, and standing positions are shown in panels A, B, and C, respectively. In each panel, the red circles and blue squares show data of individual female and male subjects, respectively. The red and blue lines with the light red and light blue bands show linear regressions in female and male subjects with their 95% confidence intervals, respectively. The light violet band shows the overlap between the confidence intervals of the regressions in female and male subjects.

https://doi.org/10.3390/diagnostics16030502

Supplementary Figure S6

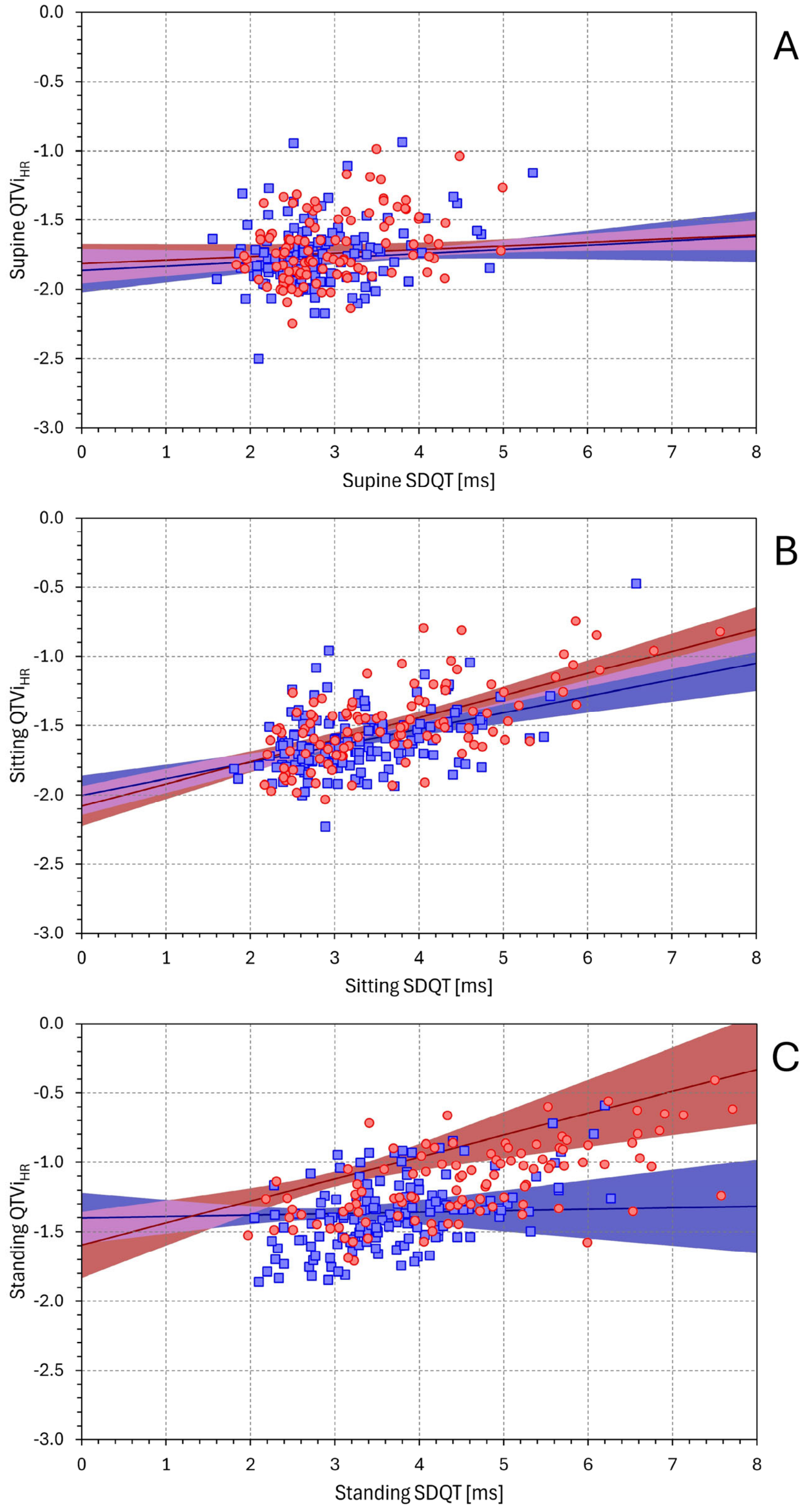

Figure S6: Relationship between SDQT and $QTVi_{HR}$ values in supine, sitting, and standing positions are shown in panels A, B, and C, respectively. In each panel, the red circles and blue squares show data of individual female and male subjects, respectively. The red and blue lines with the light red and light blue bands show linear regressions in female and male subjects with their 95% confidence intervals, respectively. The light violet band shows the overlap between the confidence intervals of the regressions in female and male subjects.

https://doi.org/10.3390/diagnostics16030502

Supplementary Figure S7

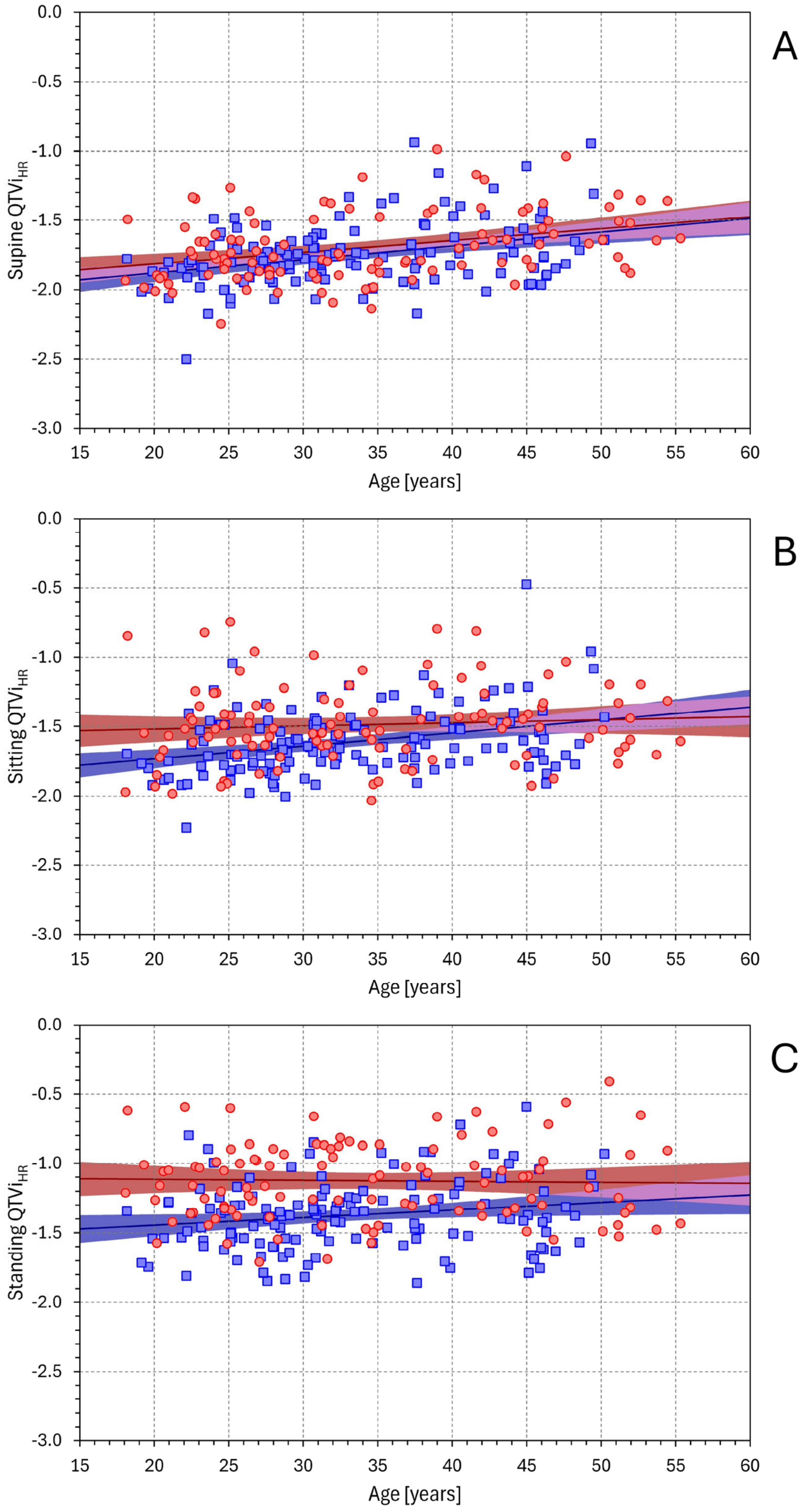

Figure S7: Relationship between Age and $QTVi_{HR}$ values in supine, sitting, and standing positions are shown in panels A, B, and C, respectively. In each panel, the red circles and blue squares show data of individual female and male subjects, respectively. The red and blue lines with the light red and light blue bands show linear regressions in female and male subjects with their 95% confidence intervals, respectively. The light violet band shows the overlap between the confidence intervals of the regressions in female and male subjects.
